# Clinical Impact of the Increase in Immunosuppressive Cell-Related Gene Expression in Urine Sediment during Intravesical Bacillus Calmette-Guérin

**DOI:** 10.3390/diseases7020044

**Published:** 2019-06-18

**Authors:** Makito Miyake, Shunta Hori, Sayuri Ohnishi, Takuya Owari, Kota Iida, Kenta Ohnishi, Yosuke Morizawa, Daisuke Gotoh, Yoshitaka Itami, Yasushi Nakai, Takeshi Inoue, Satoshi Anai, Kazumasa Torimoto, Katsuya Aoki, Tomomi Fujii, Nobumichi Tanaka, Kiyohide Fujimoto

**Affiliations:** 1 Department of Urology, Nara Medical University, 840 Shijo-cho, Kashihara-shi, Nara 634-8522, Japan; horimaus@gmail.com (S.H.); sayuri3@naramed-u.ac.jp (S.O.); tintherye@gmail.com (T.O.); kota1006ida@yahoo.co.jp (K.I.); kenzmedico0912@yahoo.co.jp (K.O.); tigers.yosuke@gmail.com (Y.M.); dgotou@gmail.com (D.G.); y.itami.324@gmail.com (Y.I.); nakaiyasusiuro@live.jp (Y.N.); you1513tt@yahoo.co.jp (T.I.); sanai@naramed-u.ac.jp (S.A.); torimoto@naramed-u.ac.jp (K.T.); aokik@naramed-u.ac.jp (K.A.); sendo@naramed-u.ac.jp (N.T.); kiyokun@naramed-u.ac.jp (K.F.); 2 Department of Diagnostic Pathology, Nara Medical University, 840 Shijo-cho, Kashihara-shi, Nara 634-8522, Japan; fujiit@naramed-u.ac.jp

**Keywords:** Bacillus Calmette-Guérin, non-muscle invasive bladder cancer, tumor-associated macrophage, regulatory T cell, myeloid-derived suppressor cell, intravesical recurrence

## Abstract

Background: The aim of this study is to evaluate the clinical impact of intravesical Bacillus Calmette-Guérin (BCG)-induced changes in blood/urinary immune markers. Methods: Time-course changes in blood/urinary clinical parameters and mRNA expression of 13 genes in urine sediment taken eight times during the treatment course of intravesical BCG (before, every 2 weeks for 8 weeks, and after) in 24 patients with non-muscle invasive bladder cancer. The genes examined include cellular markers of four immune checkpoint proteins (PD-L1, PD-L2, PD-1, and CTLA-4), immunosuppressive cells (regulatory T cells, tumor-associated macrophages, and myeloid-derived suppressor cells), pan-T lymphocytes, B lymphocytes, and neutrophils. Results: Significant transient increase in gene expression was observed for PD-L1, PD-1, FOXP3, and CD204 at 6–8 doses of BCG. The patients were stratified into two groups depending on the number of genes with increased mRNA expression. Fourteen (58%) had 0–1 genes upregulated, while 10 (42%) had 2–4 genes with increased expression. No patient in the 0–1 group experienced recurrence, while 70% of patients in the 2–4 group experienced recurrence (*p* value = 0.037, hazard ratio = 5.93). Conclusions: Our findings suggested that increases in more than one of PD-L1, PD-1, FOXP3, and CD204, expression in the urine sediments was associated with resistance to BCG treatment.

## 1. Introduction

Several guidelines including European Association of Urology (EAU) [1], American Association of Urology (AUA) [2], and The International Bladder Cancer Group (IBCG) [3] recommend intravesical instillation of Bacillus Calmette-Guérin (BCG) as adjuvant therapy for non-muscle invasive bladder cancer (NMIBC), after transurethral resection of bladder tumor (TURBT). Due to the heterogeneous clinical and biological behavior of urothelial carcinoma (UC) of the bladder, 30–50% of patients have intravesical recurrence and 15–30% experience disease progression to muscle invasive bladder cancer (MIBC) in spite of BCG treatment [4,5].

A delay in radical cystectomy (RC) leads to a shortened disease-specific survival time when compared to early RC, at the time of NMIBC [6]. Therefore, there is an urgent need for biomarkers to predict patient response to intravesical BCG. In spite of much research exploring the possible mechanisms underlying BCG-induced antitumor activity and relevant molecules [7,8,9], its biologic effects remain relatively unknown. Briefly, BCG is internalized into urothelial cells thorough complex formation with fibronectin, followed by induction of the Th1 cytokine response. Pro-inflammatory cytokines are secreted to recruit a Th1-induced immunoreaction with recognition of cancer cells through the activation and/or recruitment of mononuclear cells, polymorphonuclear neutrophils, CD8+ T-cells, and natural killer cells [5,10]. Recent evidence has demonstrated that the pre-BCG baseline status of Th1/Th2 balance, degree of regulatory T cell (Treg) recruitment, and tumor-associated macrophage (TAM) polarization in the tumor microenvironment can influence the response to intravesical BCG [5,9,11,12]. Myeloid-derived suppressor cell (MDSC) is one of the emerging key immunosuppressive cells. Two preclinical studies have shown that the reduction of MDSCs in orthotopic bladder tumors was correlated with prominent tumor growth inhibition by BCG treatment [13,14]. We hypothesized that the induction of these immunosuppressive cells during BCG treatment may attenuate the antitumor immune response, increasing the probability of intravesical recurrence and progression.

It is currently understood that the most important predictor of clinical response to BCG is a host’s ability to generate an appropriate immune response [15]. Although BCG-induced changes in the number of urinary leukocytes, levels of inflammatory cytokines in urine supernatant, and expression of inflammasome-related genes in urine sediment have been investigated [10,15,16,17,18], there is still a significant lack of studies on using inducible immunosuppressive factors to predict the clinical outcome after BCG treatment. Therefore, we conducted a prospective study to evaluate the clinical impact of intravesical BCG-induced changes on blood immune markers, urine immune cells, and expression level of immunosuppressive cell markers in urine sediment.

## 2. Materials and Methods

### 2.1. Patients Treated with Intravesical Instillation of Bacillus Calmette-Guérin

The ethics committee of the Nara Medical University approved this study, and all participants provided informed consent (reference ID: 1297). A total of 24 patients with diagnosed non-muscle invasive UC of the bladder receiving intravesical BCG after TURBT were enrolled in this study. TURBT was carried out according to a standardized procedure used by all surgeons at the single institute [4]. All patients received an induction course of intravesical BCG (BCG Tokyo 172 strain; Japan BCG Laboratory Tokyo, Japan) once weekly for 8 weeks. No patients underwent maintenance intravesical BCG. A single treatment of immediate post-TURBT chemotherapy with anthracyclines was given to a subset of the cohort. Basically, patients with suspected papillary and solitary low-grade disease were treated with immediate post-TURBT intravesical chemotherapy. All hematoxylin and eosin-stained specimens obtained from the initial TURBT were assessed by 2 experienced uropathologists (T.F.) for the T category (2010 American Joint Committee on Cancer TNM Staging system), tumor grade (2004 WHO classification), and carcinoma in situ (CIS). Follow-up was performed according to our institutional protocol [4].

### 2.2. Collection of Data and Samples

Blood and urine samples were collected before TURBT (pre-TURBT), immediately before BCG treatment (pre-BCG as the baseline), immediately before intravesical BCG at 2 doses of BCG, at 4 doses BCG, at 6 doses of BCG, and at 8 doses of BCG. After the completion of induction course, blood and urine samples were collected at 1, 3, and 6 months. In total, 200-mL of fresh voided urine samples were obtained and 10 mL of these was subjected to the standard urinalysis. An automated urine flow cytometer, Sysmex UF-1000i (Sysmex Medical Electronics Co, Kobe, Japan), was used to detect particles in urine, including white blood cells and red blood cells. The remaining urine was centrifuged at 400× *g* for 5 min at 20 °C. The supernatant was carefully decanted and the urine sediment was snap frozen. Both the supernatant and the pellet were stored at −80 °C prior to analysis.

Data collected included the level of hemoglobin, platelet count, white blood cell count and its five fractions, neutrophils, lymphocytes, monocytes, eosinophils, and basophils. Three inflammation-based markers, neutrophil-to-lymphocyte ratio (NLR), platelet-to-lymphocyte ratio (PLR), and monocyte-to-lymphocyte ratio (MLR) were calculated from the data.

### 2.3. Real-Time Reverse Transcription PCR (RT-PCR) of Urine Sediment

Peripheral blood lymphocytes from healthy volunteers were obtained as a standard control. Total RNA was extracted from urine sediment and peripheral blood lymphocytes and real-time Reverse Transcription PCR (RT-PCR) was performed, as previously described [19]. Time-course changes in mRNA expression of 13 genes in urine sediment were taken eight times during the treatment course of intravesical BCG (pre, every 2 weeks for 8 weeks, and after) in 24 patients with non-muscle invasive bladder cancer. The genes tracked included cellular markers of immune checkpoint proteins, immunosuppressive cells (Treg, TAM, and MDSC), pan-T lymphocytes, B lymphocytes, and neutrophils. Relative fold change in gene expression from the baseline (pre-BCG) was calculated after normalization by the combination of β-actin (assay ID: Hs01060665_g1) and GAPDH (assay ID: Hs02786624_g1) using the relative standard curve method. The values of peripheral blood lymphocytes were set to 1.0.

### 2.4. Statistical Analysis

The value at each treatment time point was expressed by mean ± standard deviation (SD) and median with IQR. Although all the values were normally distributed, the graphical data for the time-course changes was presented with mean ± SD for convenience. The values were compared with the pre-BCG baseline using a non-parametric test, the Wilcoxon signed-rank test. Intravesical recurrence-free survival (RFS) was estimated using the Kaplan–Meier method and compared using the log-rank test. PRISM software version 7.00 (GraphPad Software, Inc., San Diego, CA, USA) was used for statistical analysis and plotting the data. A *p* value < 0.05 was considered statistically significant.

## 3. Results

### 3.1. Patient Characteristics

Clinicopathological characteristics of the 24 patients enrolled in this study are shown in Table 1. Median age was 75 years-old (range, 58–86). Out of 24 patients, 2 (8.3%) were female, 7 (29%) had recurrent NMIBC on enrollment into this study, and 5 (21%) had concomitant CIS. After the completion of BCG induction treatment, seven (29%) experienced intravesical tumor recurrence during the follow-up period (median, 13 months). Six out of 24 patients (case nos. 4, 6, 16, 21, 23, and 24) with suspected primary or recurrent low-risk diseases were treated with immediate post-TURBT intravesical chemotherapy. Progression from Ta low-grade tumor to T1 high-grade tumor was only seen in one patient (case no. 6), who was treated with an early radical cystectomy.

### 3.2. Changes in Blood and Urine Clinical Parameters

**Figure 1:**
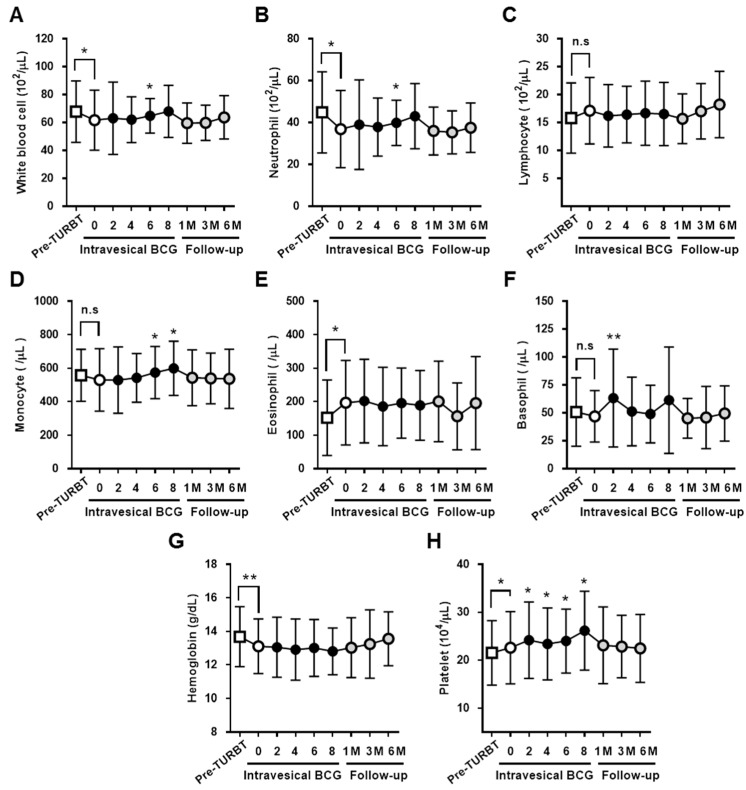
Time-course changes in clinical blood parameters in patients with non-muscle invasive bladder cancer before, during, and after the treatment. The values of blood white blood cells (**A**), neutrophils (**B**), lymphocytes (**C**), monocytes (**D**), eosinophils (**E**), basophils (**F**), hemoglobin (**G**), and platelets (**H**). The value at each treatment time point is mean ± standard deviation and compared with those at the pre-BCG baseline (indicated by ‘0’) using the Wilcoxon signed-rank test. * *p* < 0.05, ** *p* < 0.01.

Time-course changes in blood and urine clinical parameters before, during, and after intravesical BCG are summarized in Table 2. Line plots of the time-course changes are included as Figure 1 and supplementary Figures S1 and S2. Significant increases in the blood counts of neutrophils, monocytes, basophils, and platelets were observed after intravesical BCG. The neutrophil, monocyte, and platelet counts peak at 6–8 doses of intravesical BCG. The increased values had returned to baseline levels within 1 month of the completion of intravesical BCG. No significant change was observed in the urine counts of white and red blood cells during intravesical BCG, partly because the pre-BCG baseline values were high (average, 419 and 83 cells/µL, respectively). One month after the completion of intravesical BCG, there was a significant decrease in urine white and red blood cell counts, but they remained positive even at 6 months post-treatment.

### 3.3. Changes in mRNA Expression of Immune Cell Markers in Urine Sediment

**Figure 2:**
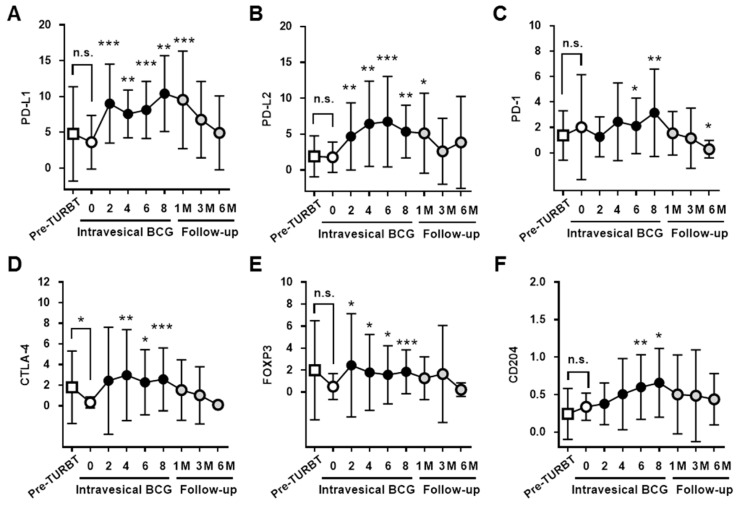
Time-course changes in mRNA expression of four immune checkpoint proteins, reguratory T cell (Treg) marker, and tumor-associated macrophage (TAM) marker in voided urine sediment from NMIBC patients before, during, and after the treatment. The expression levels of PD-L1 (**A**), PD-L2 (**B**), PD-1 (**C**), CTLA-4 (**D**), FOXP3 (**E**), and CD204 (**F**) in voided urine sediment. The value at each treatment time point is the mean ± SD and compared with those at the pre-BCG baseline (indicated by ‘0’) using the Wilcoxon signed-rank test. Changes in other parameters are shown in supplementary Figures S1–S5. * *p* < 0.05, ** *p* < 0.01, *** *p* < 0.001.

Time-course changes in the mRNA expression of immune cell markers in voided urine sediment before, during, and after intravesical BCG are summarized in Table 3. Line plots of the time-course changes are found in Figure 2 and supplementary Figures S3 and S4. A significant increase in the expression of all four immune check point proteins, Forkhead box protein 3 (FOXP3), and CD204 was observed after commencement of intravesical BCG. Their expression peaked at 6–8 doses of intravesical BCG. The increased expression had returned to baseline levels by 1–3 months after the completion of intravesical BCG. CD3E, a marker of pan-T lymphocyte, still showed a significant increase in expression even 6 months after the completion of BCG. In contrast, the markers for MDSC, B lymphocytes, and neutrophils did not show any significant change during treatment.

### 3.4. Prognostic Role for Increased mRNA Expression of Immune Suppressive Markers in NMIBC Patients Treated with Intravesical BCG

We hypothesized that increased levels of immune suppressive gene expression after commencement of BCG would be associated with a clinical outcome, namely intravesical recurrence-free survival. A transient increase was found for many parameters after intravesical BCG (Table 2 and Table 3). Among the parameters with a significant change, most of those showed a peak at 6–8 doses of BCG, with the majority returning to baseline values over time. We picked nine parameters which showed a significant increase at 6–8 doses of BCG. The difference between the baseline and 6 doses of BCG was calculated and indicated by ‘Δname of parameter’ (Table 4). For mRNA expression in urine sediment (relative values of peripheral blood lymphocytes from healthy volunteers), median change in ΔProgrammed death-ligand 1(PD-L1), ΔProgrammed death-ligand 2 (PD-L2), ΔProgrammed death-1(PD-1), ΔCytotoxic T-lymphocyte antigen 4 (CTLA-4), ΔFOXP3, and ΔCD204 expression were +4.2, +2.6, +0.50, +0.56, +1.20, and +0.23, respectively. The cutoff points for change after BCG were determined using the median values for the parameters. The gene-specific TaqMan primer and probe sets used in this study are shown in Table 3. Although no parameter was associated with a significant change in intravesical recurrence, of the six immunosuppressive genes tested in this study, higher levels of ΔPD-L1, ΔPD-1, ΔFOXP3, and ΔCD204 expression showed a negative impact on favorable clinical outcome (Table 4). Given the association between elevated levels gene expression and clinical outcome, NMIBC patients treated with intravesical BCG were stratified into two groups based on the number of genes with increased mRNA expression: 0–1 and 2–4. Of the 24 patients, 14 (58%) had 0–1 and 10 (42%) had 2–4 genes with increased expression. No patients who had none or one upregulated gene experienced intravesical recurrence, while 70% of patients having 2–4 upregulated genes experienced at least one recurrence. The CEUTO recurrence scoring model did not show the better stratification compared to the profile of four upregulated genes (Table 3).

## 4. Discussion

The present study demonstrates the association between poor clinical outcomes and a transient increase in immunosuppressive cell-related gene expression in urine sediment during intravesical BCG. Since intravesical BCG was introduced in 1976 [20], no molecular biomarkers for predicting response to the treatment have been widely accepted in the clinical setting. To date, there is little evidence of association between resistance to intravesical BCG and immunosuppressive proteins, such as immune check point molecules, or immune suppressive cells such as Treg, TAM, and MDSC cells. We recently reported that increased counts of Treg and TAM in the baseline tumor immune environment are associated with shorter intravesical recurrence-free survival in NMIBC treated with intravesical BCG [5]. Similar results have been reported by Pichler et al. and Suriano et al. [9,12]. 

Many studies which focus on tumor markers seem to ignore the intrinsic therapeutic mechanism of intravesical BCG. A host’s capability and potential to generate an appropriate immune response are essential to achieve BCG-related clinical efficacy. Sanchez-Carbayo et al. evaluated the potential role of serial urinary interleukins (ILs) as a surveillance biomarker for treatment of NMIBC [18]. Although elevated levels of urinary cytokine IL-2 were associated with a favorable response to intravesical BCG, elevated levels of the non-specific cytokines IL-6 and IL-8 were observed due to concurrent urinary tract infections. Kamat et al. conducted a clinical trial of 130 patients with NMIBC treated with BCG according to the Southwest Oncology Group protocol [15]. Their analysis focused on the changes in urinary levels of nine out of 12 tested cytokines from pre-BCG baseline values. A nomogram constructed from the nine inducible cytokines (IL-2, IL-8, IL-6, IL-1ra, IL-10, IL-12, IL-12, TRAIL, and TNF-α) successfully predicted the recurrence rate with an accuracy of 85.5%. These results suggest the potential of using changes in inducible factors after intravesical BCG as a tool for identifying patients at risk of tumor recurrence and progression. A combination of cytokines, e.g., a panel of cytokines or nomogram, may be required for better accuracy. The recent clinical success of immune check point antagonists targeting the PD-1/PD-L1 axis and CTLA-4 provides a clear paradigm shift in drug development [21]. This development raises the possibility that monitoring immune check point molecules and/or immunosuppressive cells could also be useful tools for predicting the treatment efficacy of immunotherapies. To address the lack of tools predicting the clinical outcome of BCG treatment, we conducted a prospective clinical trial to determine the extent to which the inducible level of immunosuppressive cell markers in urine sediment during intravesical BCG predicts recurrence.

Our analysis included the post-treatment change of clinical blood and urine parameters such as white blood cells, neutrophils, platelets, and NLR. Among the parameters tested, the count of blood platelets showed the biggest increase after intravesical BCG (Table 2). There is currently a large body of evidence that platelets play central roles in inflammatory reactions [22]. Platelets not only enhance vascular permeability and leukocyte infiltration into the stromal tissue but also modulate the effector functions of neutrophils and macrophages. Because intravesical BCG is known to involve the recruitment of neutrophils and macrophages to the bladder, it is reasonable that an increase in the count of blood platelets is one of the systemic immune responses to this therapy. While a previous study suggested that baseline levels of pyuria might be an independent predictor for the success of intravesical BCG [17], clinical urinalysis showed that the counts of white and red blood cells were not significantly affected by intravesical BCG.

**Figure 3:**
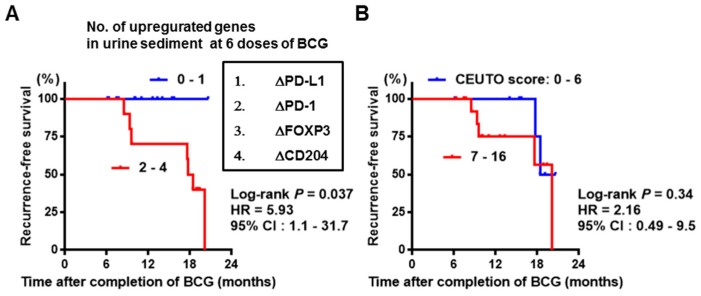
Intravesical recurrence-free survival curves. (**A**) Stratification according to the number of upregulated genes in urine sediment after intravesical BCG. The log-rank test was used for the comparison between 0–1 (n = 14) and 2–4 (n = 10). (**B**) Stratification according to the CUETO recurrence score. The log-rank test was used for the comparison between 0–6 (n = 9) and 7–16 (n = 15). HR, hazard ratio; CI, confidence interval.

Although the relationship between two immunosuppressive cells, Treg and TAM, and response to BCG treatment have been studied [5,9,12], little is known about the role of MDSC in BCG treatment outcomes. Treg plays important roles in escape from response to treatments in various malignancies, leading to poor oncological outcomes [23]. Many intensive studies have demonstrated that TAM cells are markedly present in various malignancies and are involved in promoting neoangiogenesis via the production of immunosuppressive cytokines, leading to worsened oncological outcomes [24]. MDSCs are a heterogeneous population of immature myeloid cells with an immune suppressive phenotype in various malignancies [25,26]. They exert direct effects on the innate and adaptive immune response through supporting immune escape, largely through the maintenance of a high oxidative stress environment. The recruitment of MDSC positively correlates with a diminishing effectiveness of immunotherapies, including various immune checkpoint inhibitors [25]. In the present study, we hypothesized that intravesical recurrence-free survival would be associated with the change in MDSC during BCG treatment. None of the MDSC markers, CD11b, CD14, or CD33, showed a significant increase in mRNA expression in urine sediment during intravesical BCG. This finding suggests that MDSC does not play a vital role on resistance to BCG treatment. In contrast, elevated levels of PD-L1, PD-1, FOXP3, and CD204 expression were associated with resistance to BCG treatment and a poor clinical outcome (Table 4 and Figure 3).

The present study has several limitations. The first is potential selection bias; for example, some patients were excluded due to patient refusal. Secondly, we did not perform immune cell phenotyping using flow cytometry analysis. RT-PCR analysis with a single marker is not able to determine the true immune cell population. Third, the analysis includes a limited number of patients, only 24 patients, with a relatively short follow-up duration following intravesical BCG. This did not allow for multivariate analysis to derive reliable prognostic values. Lastly, there is the lack of data from ‘controls’ (healthy individuals) to indicate that the low levels of variation in the parameters tested would not randomly occur naturally. We conducted the present pilot study to explore the value of, a future prospective study enrolling a larger sample size with a longer follow-up period.

In conclusion, we analyzed detailed time-course changes during and after intravesical BCG in clinical blood/urinary tests and urine sediment-derived mRNA expression of 10 genes with immunosuppressive roles. Moreover, we explored the prognostic role of increased mRNA expression of immune suppressive markers, suggesting that larger increases in PD-L1, PD-1, FOXP3, and CD204 expression were associated with resistance to BCG treatment and poor clinical outcome. To evaluate the clinical role of MDSC in this treatment subset, more intensive study using flow cytometry analysis may be needed. A future prospective study enrolling a larger sample size with a longer follow-up period is warranted for confirming the true clinical benefit of the biomarkers identified in this study.

## Figures and Tables

**Table 1:** Clinicopathological characteristics of 24 patients enrolled in this study.

No	Sex	Age (y.o.)	Primary or Recurrent	Tumor Stage/Grade	CIS	Tumor Size (mm)	Multiplicity	CUETO Recurrence Score	Recurrence after BCG
1	M	82	Primary	T1/LG	No	25	Multiple	9	Recurrence-free
2	M	64	Primary	Ta/LG	No	30	Solitary	2	Recurrence-free
3	M	73	Primary	Ta/LG	No	30	Solitary	3	Recurrence-free
4	F	86	Primary	Ta/LG	No	10	Solitary	6	18 months
5	M	62	Recurrent	Ta/LG	No	50	Multiple	8	Recurrence-free
6	M	83	Recurrent	Ta/LG	No	20	Solitary	7	20 months
7	M	72	Primary	T1/HG	Yes	40	Multiple	9	Recurrence-free
8	M	70	Primary	Ta/LG	No	45	Multiple	3	18 months
9	M	84	Primary	T1/HG	No	60	Multiple	7	18 months
10	M	84	Primary	T1/HG	No	50	Multiple	7	Recurrence-free
11	M	58	Primary	Ta/LG	No	30	Multiple	3	Recurrence-free
12	M	68	Primary	T1/HG	No	25	Solitary	4	Recurrence-free
13	M	79	Primary	T1/HG	No	30	Multiple	5	Recurrence-free
14	F	67	Primary	Ta/LG	No	30	Multiple	7	Recurrence-free
15	M	65	Primary	Ta/HG	Yes	30	Multiple	8	Recurrence-free
16	M	79	Recurrent	Ta/LG	No	10	Multiple	9	Recurrence-free
17	M	73	Primary	T1/HG	No	45	Multiple	7	9 months
18	M	83	Recurrent	T1/HG	Yes	30	Multiple	13	10 months
19	M	76	Recurrent	Ta/LG	No	10	Multiple	9	10 months
20	M	77	Recurrent	T1/HG	No	5	Solitary	9	Recurrence-free
21	M	74	Primary	T1/HG	Yes	30	Multiple	9	Recurrence-free
22	M	81	Recurrent	T1/HG	No	30	Multiple	11	Recurrence-free
23	M	64	Primary	Ta/HG	Yes	40	Solitary	4	Recurrence-free
24	M	85	Primary	Ta/LG	No	20	Solitary	3	Recurrence-free

CIS, carcinoma in situ; CUETO, the Spanish Urological Club for Oncological Treatment; BCG, Bacillus de Calmette-Guérin; LG, low grade; HG, high grade.

**Table 2:** Time-course changes in blood and urine clinical parameters before, during, and after intravesical BCG.

Blood and Urine Clinical Parameter		Pre-TURBT	Pre-BCG (Baseline)	During BCG Treatment				After BCG		
				2 Weeks	4 Weeks	6 Weeks	8 Weeks	1 Month	3 Months	6 Months
White blood cell (×10^2^/μL)	Mean ± SD	68 ± 22	62 ± 22	63 ± 26	62 ± 16	65 ± 12	68 ± 19	60 ± 14	60 ± 13	64 ± 16
	Median (IQR)	68 (50–80)	61 (47–71)	59 (49–71)	64 (53–75)	65 (58–72)	65 (53–83)	61 (51–69)	61 (52–66)	65 (49–74)
	*p* value (vs. baseline)	0.045	baseline	0.78	0.49	0.047	0.26	0.82	0.57	0.71
Neutrophil (×10^2^/μL)	Mean ± SD	45 ± 19	37 ± 19	39 ± 21	38 ± 14	40 ± 11	43 ± 16	36 ± 12	35 ± 10	38 ± 12
	Median ± IQR	43 (29-53)	33 (27–43)	34 (27–45)	35 (29–49)	38 (33–48)	44 (34–53)	36 (26–43)	36 (29–43)	40 (27–47)
	*p* value (vs. baseline)	0.011	baseline	0.43	0.3	0.033	0.12	0.96	0.49	0.81
Lymphocyte (×10^2^/μL)	Mean ± SD	16 ± 6.3	17 ± 6	16 ± 5.6	16 ± 5.1	17 ± 5.7	17 ± 5.7	16 ± 4.5	17 ± 5	18 ± 6
	Median (IQR)	16 (12–18)	16 (14–21)	16 (11–19)	16 (13–20)	17 (13–19)	16 (12–19)	16 (13–18)	17 (13–20)	17 (14–24)
	*p* value (vs. baseline)	0.12	baseline	0.14	0.14	0.45	0.31	0.13	0.66	0.71
Monocyte (/μL)	Mean ± SD	558 ± 155	530 ± 187	529 ± 198	543 ± 146	574 ± 156	599 ± 162	544 ± 167	539 ± 151	537 ± 176
	Median ± IQR	545 (456–658)	478 (429–590)	510 (387–622)	515 (424–662)	517 (463–666)	619 (442–740)	484 (416–686)	517 (410–650)	527 (399–604)
	*p* value (vs. baseline)	0.6	baseline	0.86	0.29	0.031	0.016	0.33	0.12	0.39
Eosinophiil (/μL)	Mean ± SD	152 ± 113	197 ± 126	202 ± 125	186 ± 117	196 ± 104	189 ± 104	201 ± 120	157 ± 100	196 ± 139
	Median (IQR)	120 (78–238)	154 (117–253)	172 (104–269)	171 (106–233)	170 (121–269)	149 (114–264)	149 (116–313)	141 (91–210)	162 (133–230)
	*p* value (vs. baseline)	0.029	baseline	0.93	0.35	0.69	0.25	0.83	0.47	0.24
Basophil (/μL)	Mean ± SD	51 ± 31	47 ± 23	63 ± 44	51 ± 31	49 ± 26	62 ± 48	45 ± 18	46 ± 28	50 ± 25
	Median (IQR)	46 (26–67)	41 (31–57)	53 (35–72)	44 (33–51)	43 (32–63)	51 (42–67)	41 (35–49)	39 (31–49)	44 (31–65)
	*p* value (vs. baseline)	0.14	baseline	0.006	0.21	0.99	0.2	0.92	0.31	0.6
Hemoblobin (g/dL)	Mean ± SD	14 ± 1.8	13 ± 1.6	13 ± 1.8	13 ± 1.8	13 ± 1.7	13 ± 1.4	13 ± 1.8	13 ± 2	14 ± 1.6
	Median (IQR)	14 (13–15)	13 (13–14)	13 (12–14)	13 (12–14)	13 (12–14)	13 (12–14)	13 (12–14)	14 (13–14)	14 (13–15)
	*p* value (vs. baseline)	0.006	baseline	0.86	0.28	0.3	0.19	0.74	0.33	0.78
Platelet (×10^4^/μL)	Mean ± SD	22 ± 6.8	23 ± 7.5	24 ± 8	23 ± 7.5	24 ± 6.7	26 ± 8.2	23 ± 8.0	23 ± 6.5	22 ± 7.1
	Median (IQR)	21 (17–23)	21 (18–24)	21 (19–26)	21 (19–26)	22 (19–28)	24 (21–30)	21 (17–25)	22 (18–27)	21 (18–24)
	*p* value (vs. baseline)	0.028	baseline	0.034	0.036	0.022	0.017	0.91	0.98	0.45
NLR	Mean ± SD	74 ± 108	83 ± 130	59 ± 92	52 ± 115	45 ± 73	32 ± 57	53 ± 80	19 ± 26	28 ± 40
	Median (IQR)	32 (2.7–135)	41 (16–93)	13 (4.6–76)	20 (5.1–41)	18 (5.3–70)	18 (2.8–31)	21 (6.5–58)	9 (3.9–22)	12 (4.9–33)
	*p* value (vs. baseline)	0.023	baseline	0.53	0.33	0.078	0.11	0.79	0.85	0.71
PLR	Mean ± SD	153 ± 77	152 ± 80	166 ± 81	156 ± 70	160 ± 68	179 ± 93	162 ± 78	150 ± 80	134 ± 57
	Median (IQR)	136 (120–156)	123 (95–178)	149 (104–182)	143 (104–174)	136 (118–191)	165 (118–190)	142 (99–184)	132 (98–163)	120 (98–152)
	*p* value (vs. baseline)	0.29	baseline	0.019	0.11	0.17	0.034	0.61	0.59	0.45
MLR	Mean ± SD	0.43 ± 0.29	0.36 ± 0.2	0.36 ± 0.17	0.37 ± 0.19	0.39 ± 0.22	0.41 ± 0.21	0.38 ± 0.18	0.35 ± 0.17	0.31 ± 0.11
	Median ± IQR	0.35 (0.23–0.51)	0.29 (0.23–0.44)	0.31 (0.23–0.48)	0.32 (0.27–0.44)	0.35 (0.26–0.45)	0.36 (0.26–0.48)	0.33 (0.26–0.42)	0.3 (0.24–0.37)	0.27 (0.22–0.41)
	*p* value (vs. baseline)	0.15	baseline	0.94	0.23	0.095	0.11	0.43	0.92	0.86
Urine white blood cell (/μL)	Mean ± SD	104 ± 183	419 ± 640	327 ± 514	362 ± 779	463 ± 601	369 ± 446	232 ± 463	133 ± 235	17 ± 20
	Median (IQR)	36 (1.5–133)	224 (62–413)	152 (34–335)	158 (70–266)	198 (61–622)	120 (51–567)	59 (18–206)	7.2 (2.5–220)	7 (3.4–27)
	*p* value (vs. baseline)	0.1	baseline	0.43	0.32	0.99	0.54	0.018	0.013	0.001
Urine red blood cell (/μL)	Mean ± SD	74 ± 108	83 ± 130	59 ± 92	52 ± 115	45 ± 73	32 ± 57	53 ± 80	19 ± 26	28 ± 40
	Median (IQR)	32 (2.7–135)	41 (16–93)	13 (4.6–76)	20 (5.1–41)	18 (5.3–70)	18 (2.8–31)	21 (6.5–58)	9 (3.9–22)	12 (4.9–33)
	*p* value (vs. baseline)	0.73	baseline	0.17	0.14	0.076	0.31	0.54	0.011	0.15

TURBT, transurethral resection of bladder tumor; BCG, Bacillus Calmette-Guérin; SD, standard deviation; IQR, interquartile range; NLR, neutrophil-to-lymphocyte ratio; PLR, platelet-to-lymphocyte ratio; MLR, monocyte-to-lymphocyte ratio; *p* values are based on the comparison of the values at each treatment time point relative to the pre-BCG baseline with Wilcoxon signed-rank test. Statistical significance as compared to the pre-BCG value (baseline) is indicated by red letters.

**Table 3:** Time-course changes in mRNA expression of immune cell markers in urine sediments before, during, and after intravesical BCG.

Target	RNA Expression in Urine Sediments (TaqMan Assay ID)		Pre-TURBT	Pre-BCG (Baseline)	During BCG Treatment				After BCG		
					2 Weeks	4 Weeks	6 Weeks	8 Weeks	1 Month	3 Months	6 Months
Immune checkpoint	PD-L1 (CD274)	Mean ± SD	4.8 ± 6.6	3.6 ± 3.8	9.0 ± 5.5	7.6 ± 3.3	8.1 ± 4.0	10 ± 5.3	9.5 ± 6.8	6.8 ± 5.3	4.9 ± 5.2
	(assay ID: Hs00204257_m1)	Median (IQR)	2.2 (0.96–4.6)	2.8 (1.5–4.3)	8.8 (4.7–13)	7.6 (6–9.2)	7.2 (4.6–12)	9.6 (6–15)	9.2 (4.5–12)	5.3 (2.7–9.4)	4.8 (0.89–6.1)
		*p* value	0.96	baseline	0.0001	0.0012	0.0006	0.0034	0.0001	0.065	0.33
	PD-L2 (CD273)	Mean ± SD	1.9 ± 2.9	1.8 ± 2.1	4.7 ± 4.7	6.4 ± 5.9	6.7 ± 6.3	5.4 ± 3.7	5.1 ± 5.6	2.6 ± 4.6	3.8 ± 6.4
	(assay ID: Hs00228839_m1)	Median (IQR)	0.68 (0–2.3)	1.3 (0–3)	4 (1.9–6.1)	3.7 (3.1–9.4)	4 (3–8.8)	4.8 (1.9–7.2)	4 (1.7–6.3)	0 (0–4.2)	1.3 (0–4.9)
		*p* value	0.68	baseline	0.0043	0.0012	0.0006	0.0084	0.027	0.76	0.54
	PD-1	Mean ± SD	1.4 ± 2	2 ± 4.1	1.3 ± 1.6	2.4 ± 3.1	2.1 ± 2.2	3.2 ± 3.4	1.5 ± 1.7	1.1 ± 2.4	0.28 ± 0.69
	(assay ID: Hs01550088_m1)	Median (IQR)	0.24 (0.082–2.4)	0.69 (0–1.7)	0.76 (0.07–1.7)	1.1 (0.079–3.6)	1.4 (0.32–3)	1.6 (0.4–6.5)	1 (0.076–2.2)	0.092 (0–0.97)	0 (0–0.11)
		*p* value	0.53	baseline	0.8	0.026	0.04	0.003	0.74	0.59	0.024
	CTLA4	Mean ± SD	1.8 ± 3.5	0.34 ± 0.54	2.4 ± 5.2	3 ± 4.4	2.3 ± 3.2	2.6 ± 3.1	1.5 ± 2.9	1 ± 2.8	0.12 ± 0.28
	(assay ID: Hs00175480_m1)	Median (IQR)	1.9 (0.019–11)	0.71 (0.078–2.3)	2.7 (0.58–23)	6.4 (0.14–15)	3.9 (0.56–9.4)	6 (1.5–8.9)	1.5 (0.55–13)	0.18 (0–11)	0.053 (0–1)
		*p* value	0.36	baseline	0.057	0.0077	0.012	0.0002	0.051	0.76	0.16
Reguratory T cell	FOXP3	Mean ± SD	2 ± 4.5	0.51 ± 1.2	2.4 ± 4.7	1.8 ± 3.5	1.6 ± 2.6	1.9 ± 2.0	1.3 ± 2.0	1.7 ± 4.4	0.24 ± 0.61
(Treg)	(assay ID: Hs01085834_m1)	Median (IQR)	0.009 (0–1.2)	0.02 (0–0.6)	0.075 (0–2.9)	0.11 (0–2.8)	0.22 (0–1.9)	1.2 (0.14–3.3)	0.26 (0–2.7)	0 (0–1.5)	0 (0–0.11)
		*p* value	0.5	baseline	0.048	0.048	0.042	0.0005	0.083	0.76	0.28
	ISG20 (CD25)	Mean ± SD	1.7 ± 1.8	1.2 ± 0.72	1.3 ± 0.76	1.5 ± 0.91	1.6 ± 1.4	1.8 ± 1.1	1.5 ± 1.1	1.1 ± 0.83	0.76 ± 0.46
	(assay ID: Hs00158122_m1)	Median ± IQR	1.1 (0.67–1.9)	1.1 (0.71–1.8)	1.2 (0.8–1.8)	1.3 (0.73–2.1)	1 (0.85–2.1)	1.6 (1.1–1.9)	1.1 (0.74–1.7)	0.89 (0.5–1.4)	0.65 (0.31–1.1)
		*p* value	0.99	baseline	0.56	0.34	0.27	0.28	0.65	0.86	0.23
Tumor-associated macrophage	MSR1 (CD204)	Mean ± SD	0.24 ± 0.34	0.34 ± 0.18	0.38 ± 0.28	0.51 ± 0.48	0.6 ± 0.43	0.66 ± 0.46	0.5 ± 0.53	0.49 ± 0.61	0.44 ± 0.34
(TAM)	(assay ID: Hs00234007_m1)	Median (IQR)	0.091 (0.04–0.32)	0.29 (0.23–0.44)	0.3 (0.14–0.53)	0.3 (0.17–0.69)	0.52 (0.24–0.88)	0.67 (0.33–0.74)	0.41 (0.16–0.58)	0.26 (0.071–0.66)	0.38 (0.12–0.65)
		*p* value	0.08	baseline	0.63	0.13	0.009	0.011	0.65	0.98	0.055
Myeloid-derived suppressor cells	ITGAM (CD11b)	Mean ± SD	0.23 ± 0.32	0.098 ± 0.088	0.14 ± 0.12	0.14 ± 0.13	0.26 ± 0.45	0.25 ± 0.54	0.096 ± 0.16	0.088 ± 0.11	0.072 ± 0.14
(MDSC)	(assay ID: Hs00167304_m1)	Median (IQR)	0.083 (0.021–0.33)	0.072 (0.026–0.18)	0.091 (0.035–0.21)	0.097 (0.028–0.26)	0.07 (0.033–0.28)	0.071 (0.022–0.28)	0.032 (0.021–0.098)	0.036 (0.008–0.13)	0.036 (0.021–0.056)
		*p* value	0.75	baseline	0.41	0.26	0.41	0.64	0.62	0.46	0.055
	CD14	Mean ± SD	2.7 ± 3.5	3 ± 3.6	2.4 ± 2.3	2.1 ± 1.9	1.8 ± 1.6	1.4 ± 1.4	3.4 ± 4.6	4.3 ± 5.5	4.8 ± 7.23
	(assay ID: Hs02621496_s1)	Median (IQR)	1.4 (0.56–3.4)	1.5 (0.91–3.5)	1.6 (0.9–3.1)	1.4 (0.84–2.3)	1.1 (0.69–2.5)	1.1 (0.57–1.6)	2.5 (1.45–3.8)	1.6 (0.8–5.9)	2.5 (1.5–5.2)
		*p* value	0.46	baseline	0.99	0.61	0.17	0.33	0.68	0.35	0.33
	CD33	Mean ± SD	0.064 ± 0.058	0.11 ± 0.079	0.13 ± 0.11	0.14 ± 0.099	0.15 ± 0.11	0.11 ± 0.086	0.14 ± 0.13	0.14 ± 0.13	0.21 ± 0.24
	(assay ID: Hs01076282_g1)	Median (IQR)	0.045 (0.015–0.1)	0.085 (0.049–0.2)	0.11 (0.061–0.14)	0.12 (0.08–0.18)	0.13 (0.091–0.18)	0.099 (0.031–0.15)	0.1 (0.061–0.18)	0.084 (0.029–0.25)	0.13 (0.028–0.38)
		*p* value	0.027	baseline	0.79	0.23	0.17	0.45	0.33	0.64	0.33
Pan-T lymphocyte	CD3E	Mean ± SD	0.097 ± 0.15	0.024 ± 0.044	0.069 ± 0.099	0.12 ± 0.18	0.11 ± 0.15	0.18 ± 0.19	0.16 ± 0.17	0.085 ± 0.13	0.1 ± 0.16
	(assay ID: Hs01062241_m1)	Median (IQR)	0.021 (0.006–0.18)	0.009 (0.0013–0.036)	0.029 (0.004–0.093)	0.026 (0.004–0.2)	0.035 (0.016–0.15)	0.14 (0.023–0.25)	0.08 (0.016–0.34)	0.027 (0–0.12)	0.02 (0–0.17)
		*p* value	0.18	baseline	0.018	0.0024	0.002	0.0001	0.001	0.15	0.048
B lymphocyte	MS4A4A (CD20)	Mean ± SD	0.77 ± 0.19	1.7 ± 3.2	1.6 ± 1.9	4.9 ± 6.5	6.9 ± 8.6	12 ± 15	1.2 ± 1.2	1.6 ± 0.072	1.2 ± 0.97
	(assay ID: Hs00544819_m1)	Median (IQR)	0.77 (0.64–0.9)	0.12 (0–3.3)	0.6 (0.18–3.4)	0.46 (0.089–13)	1.8 (1.1–13)	5.7 (0.43–29)	0.97 (0.21–2)	1.6 (1.5–1.6)	1.2 (0.47–1.8)
		*p* value	0.11	baseline	0.81	0.11	0.016	0.06	0.99	0.99	0.99
Neutrophil	Elastase-1	Mean ± SD	2 ± 2.9	4 ± 3.9	3.4 ± 3.5	3.4 ± 3.7	2.9 ± 2.4	2.4 ± 1.9	2.5 ± 2.9	1.8 ± 3.4	1.3 ± 1.5
	(assay ID: Hs00236952_m1)	Median (IQR)	0.69 (0.45–2.8)	3.7 (1.2–5.8)	2.8 (1.1–3.6)	2.3 (0.38–5.9)	2 (0.84–4.9)	2.2 (0.56–3.7)	1.5 (0.66–2.9)	0.42 (0–2.1)	0.82 (0–2.5)
		*p* value	0.051	baseline	0.23	0.27	0.096	0.064	0.054	0.034	0.004

TURBT, transurethral resection of bladder tumor; BCG, Bacillus Calmette-Guérin; SD, standard deviation; IQR, interquartile range; NLR, neutrophil-to-lymphocyte ratio; PLR, platelet-to-lymphocyte ratio; MLR, monocyte-to-lymphocyte ratio; The values of peripheral blood lymphocytes were set as 1.0. *p* values are based on the comparison of the values at each treatment time point relative to the pre-BCG baseline with Wilcoxon signed-rank test. Statistical significance as compared to the pre-BCG value (baseline) is indicated by red letters.

**Table 4:** The prognostic factors for recurrence in 24 patients treated with intravesical BCG.

Parameters	Difference of Values [(6 Times of BCG)-(Pre-BCG)]	Intravesical Recurrence-Free Survival		
		Univariate		
		HR	95% CI	*p*-Value ^†^
neutrophils in blood	(median: +3 × 10^2^/μL)			
	≤3	1		
	>3	2.7	0.56–12.9	0.16
monocytes in blood	(median: +30/μL)			
	≤30	1		
	>30	1.3	0.28–6.2	0.69
platelets in blood	(median: +1.4 × 10^4^/μL)			
	≤1.4	1		
	>1.4	1.2	0.25–5.3	0.84
PD-L1 expression in urine sediment	(median: +4.2)			
	≤4.2	1		
	>4.2	2.2	0.2–11.8	0.22
PD-L2 expression in urine sediment	(median: +2.6)			
	≤2.6	1		
	>2.6	0.55	0.11–2.8	0.41
PD-1 expression in urine sediment	(median: +0.50)			
	≤0.50	1		
	>0.50	1.8	0.27–11.5	0.25
CTLA-4 expression in urine sediment	(median: +0.56)			
	≤0.56	1		
	>0.56	0.56	0.08–3.8	0.39
FOXP3 expression in urine sediment	(median: +0.20)			
	≤0.20	1		
	>0.20	1.60	0.24–9.7	0.31
CD204 expression in urine sediment	(median: +0.23)			
	≤0.23	1		
	>0.23	2.78	0.56–10.9	0.22

NMIBC, non-muscle invasive bladder cancer; HR, hazard ratio; CI, confidence interval, ^†^ estimated using the Kaplan–Meier method and compared using the log-rank test.

## Supplementary Materials

The following are available online at https://www.mdpi.com/2079-9721/7/2/44/s1, Figure S1: Time-course changes in blood inflammation markers in NMIBC patients before, during and after the treatment, Figure S2: Time-course changes in urianalysis in NMIBC patients before, during and after the treatment, Figure S3: Time-course changes in mRNA expression of MDSC markers in voided urine sediment from NMIBC patients before, during and after the treatment, Figure S4: Time-course changes in mRNA expression of pan-T lymphocyte, B lymphocyte, and neutrophil makers in voided urine sediment from NMIBC patients before, during and after the treatment. The value at each treatment time point is mean ± standard deviation and compared with those at the pre-BCG baseline (indicated by ‘0’) using the Wilcoxon signed-rank test. * *p* < 0.05, ** *p* < 0.01, *** *p* < 0.001.

## References

[1] Witjes J.A., Compérat E., Cowan N.C., De Santis M., Gakis G., Lebret T., Ribal M.J., Van der Heijden A.G., Sherif A., European Association of Urology (2014). EAU guidelines on muscle-invasive and metastatic bladder cancer: Summary of the 2013 guidelines. Eur. Urol..

[2] Hall M.C., Chang S.S., Dalbagni G., Pruthi R.S., Seigne J.D., Skinner E.C., Wolf J.S., Schellhammer P.F. (2007). Guidelines for the management of nonmuscle invasive bladder cancer (stages Ta, T1, and Tis): 2007 update. J. Urol..

[3] Brausi M., Witjes J.A., Lamm D., Persad R., Palou J., Colombel M., Buckley R., Soloway M., Akaza H., Böhle A. (2011). A review of current guidelines and best practice recommendations for the management of nonmuscle invasive bladder cancer by the International Bladder Cancer Group. J. Urol..

[4] Miyake M., Gotoh D., Shimada K., Tatsumi Y., Nakai Y., Anai S., Torimoto K., Aoki K., Tanaka N., Konishi N. (2015). Exploration of risk factors predicting outcomes for primary T1 high-grade bladder cancer and validation of the Spanish Urological Club for Oncological Treatment scoring model: Long-term follow-up experience at a single institute. Int. J. Urol..

[5] Miyake M., Tatsumi Y., Gotoh D., Ohnishi S., Owari T., Iida K., Ohnishi K., Hori S., Morizawa Y., Itami Y. (2017). Regulatory T cells and tumor-associated macrophages in the tumor microenvironment in non-muscle invasive bladder cancer treated with intravesical Bacille Calmette-Guérin: A long-term follow-up study of a Japanese cohort. Int. J. Mol. Sci..

[6] Raj G.V., Herr H., Serio A.M., Donat S.M., Bochner B.H., Vickers A.J., Dalbagni G. (2007). Treatment paradigm shift may improve survival of patients with high risk superficial bladder cancer. J. Urol..

[7] Kitamura H., Tsukamoto T. (2011). Immunotherapy for urothelial carcinoma: Current status and perspectives. Cancers.

[8] Abebe F. (2012). Is interferon-gamma the right marker for bacilli Calmette-Guérin-induced immune protection? The missing link in our understanding of tuberculosis immunology. Clin. Exp. Immunol..

[9] Pichler R., Fritz J., Zavadil C., Schäfer G., Culig Z., Brunner A. (2016). Tumor-infiltrating immune cell subpopulations influence the oncologic outcome after intravesical Bacillus Calmette-Guérin therapy in bladder cancer. Oncotarget.

[10] Siracusano S., Vita F., Abbate R., Ciciliato S., Borelli V., Bernabei M., Zabucchi G. (2007). The role of granulocytes following intravesical BCG prophylaxis. Eur. Urol..

[11] Nunez-Nateras R., Castle E.P., Protheroe C.A., Stanton M.L., Ocal T.I., Ferrigni E.N., Ochkur S.I., Jacobsen E.A., Hou Y.X., Andrews P.E. (2014). Predicting response to Bacillus Calmette-Guérin (BCG) in patients with carcinoma in situ of the bladder. Urol. Oncol..

[12] Suriano F., Santini D., Perrone G., Amato M., Vincenzi B., Tonini G., Muda A., Boggia S., Buscarini M., Pantano F. (2013). Tumor associated macrophages polarization dictates the efficacy of BCG instillation in non-muscle invasive urothelial bladder cancer. J. Exp. Clin. Cancer Res..

[13] Wang Y., Liu J., Yang X., Liu Y., Liu Y., Li Y., Sun L., Yang X., Niu H. (2018). Bacillus Calmette-Guérin and anti-PD-L1 combination therapy boosts immune response against bladder cancer. Onco Targets Ther..

[14] Huang P., Ma C., Xu P., Guo K., Xu A., Liu C. (2015). Efficacy of intravesical Bacillus Calmette-Guérin therapy against tumor immune escape in an orthotopic model of bladder cancer. Exp. Ther. Med..

[15] Kamat A.M., Briggman J., Urbauer D.L., Svatek R., Nogueras González G.M., Anderson R., Grossman H.B., Prat F., Dinney C.P. (2016). Cytokine panel for response to intravesical therapy (CyPRIT): Nomogram of changes in urinary cytokine levels predicts patient response to Bacillus Calmette-Guérin. Eur. Urol..

[16] Poli G., Cochetti G., Boni A., Egidi M.G., Brancorsini S., Mearini E. (2017). Characterization of inflammasome-related genes in urine sediments of patients receiving intravesical BCG therapy. Urol. Oncol..

[17] Azuma T., Nagase Y., Oshi M. (2015). Pyuria predicts poor prognosis in patients with non-muscle-invasive bladder cancer treated with bacillus Calmette-Guérin. Mol. Clin. Oncol..

[18] Sanchez-Carbayo M., Urrutia M., Romani R., Herrero M., Gonzalez de Buitrago J.M., Navajo J.A. (2001). Serial urinary IL-2, IL-6, IL-8, TNFalpha, UBC, CYFRA 21-1 and NMP22 during follow-up of patients with bladder cancer receiving intravesical BCG. Anticancer Res..

[19] Morales A., Eidinger D., Bruce A.W. (1976). Intracavitary Bacillus Calmette-Guerin in the treatment of superficial bladder tumors. J. Urol..

[20] Emens L.A., Butterfield L.H., Hodi FSJr Marincola F.M., Kaufman H.L. (2016). Cancer immunotherapy trials: Leading a paradigm shift in drug development. J. Immunother. Cancer.

[21] Gros A., Ollivier V., Ho-Tin-Noé B. (2015). Platelets in inflammation: Regulation of leukocyte activities and vascular repair. Front. Immunol..

[22] Hinz S., Pagerols-Raluy L., Oberg H.H., Ammerpohl O., Grüssel S., Sipos B., Grützmann R., Pilarsky C., Ungefroren H., Saeger H.D. (2007). Foxp3 expression in pancreatic carcinoma cells as a novel mechanism of immune evasion in cancer. Cancer Res..

[23] Pollard J.W. (2004). Tumour-educated macrophages promote tumour progression and metastasis. Nat. Rev. Cancer.

[24] Liu Y., Wei G., Cheng W.A., Dong Z., Sun H., Lee V.Y., Cha S.C., Smith D.L., Kwak L.W., Qin H. (2018). Targeting myeloid-derived suppressor cells for cancer immunotherapy. Cancer Immunol. Immunother..

[25] Ma J., Xu H., Wang S. (2018). Immunosuppressive role of myeloid-derived suppressor cells and therapeutic targeting in lung cancer. J. Immunol. Res..

[26] Miyake M., Hori S., Morizawa Y., Tatsumi Y., Nakai Y., Anai S., Torimoto K., Aoki K., Tanaka N., Shimada K. (2016). CXCL1-mediated interaction of cancer cells with tumor-associated macrophages and cancer-associated fibroblasts promotes tumor progression in human bladder cancer. Neoplasia.

