# Supplementary figures and images for: Clinical Impact of the Increase in Immunosuppressive Cell-Related Gene Expression in Urine Sediment during Intravesical Bacillus Calmette-Guérin
Source: Diseases. 2019 Jun 18;7(2):44. doi: 10.3390/diseases7020044 (PMC6630414; doi:10.3390/diseases7020044)

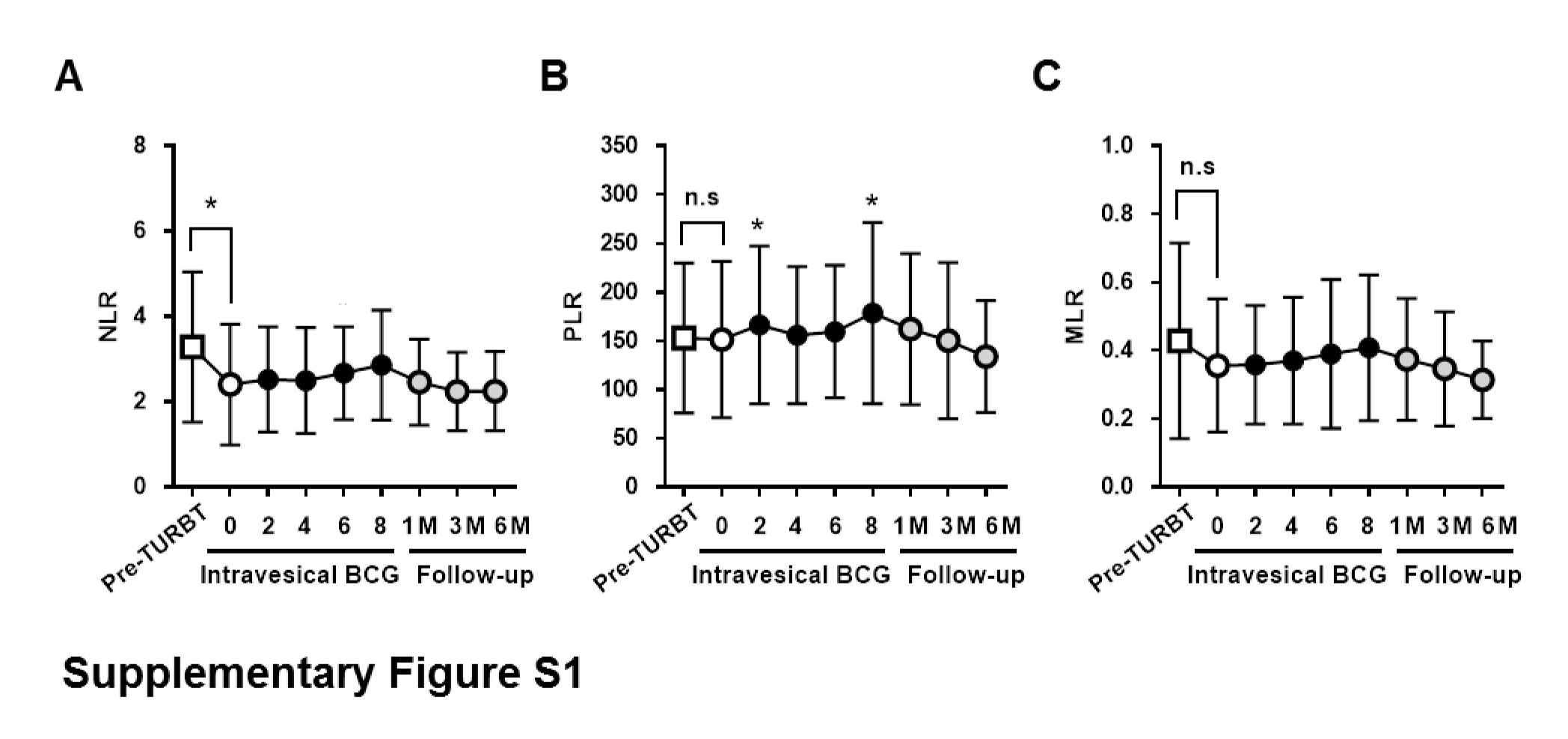

Supplement: Supplementary file 1 [file diseases-07-00044-s001.zip › Supplementary Figure S1.tif]

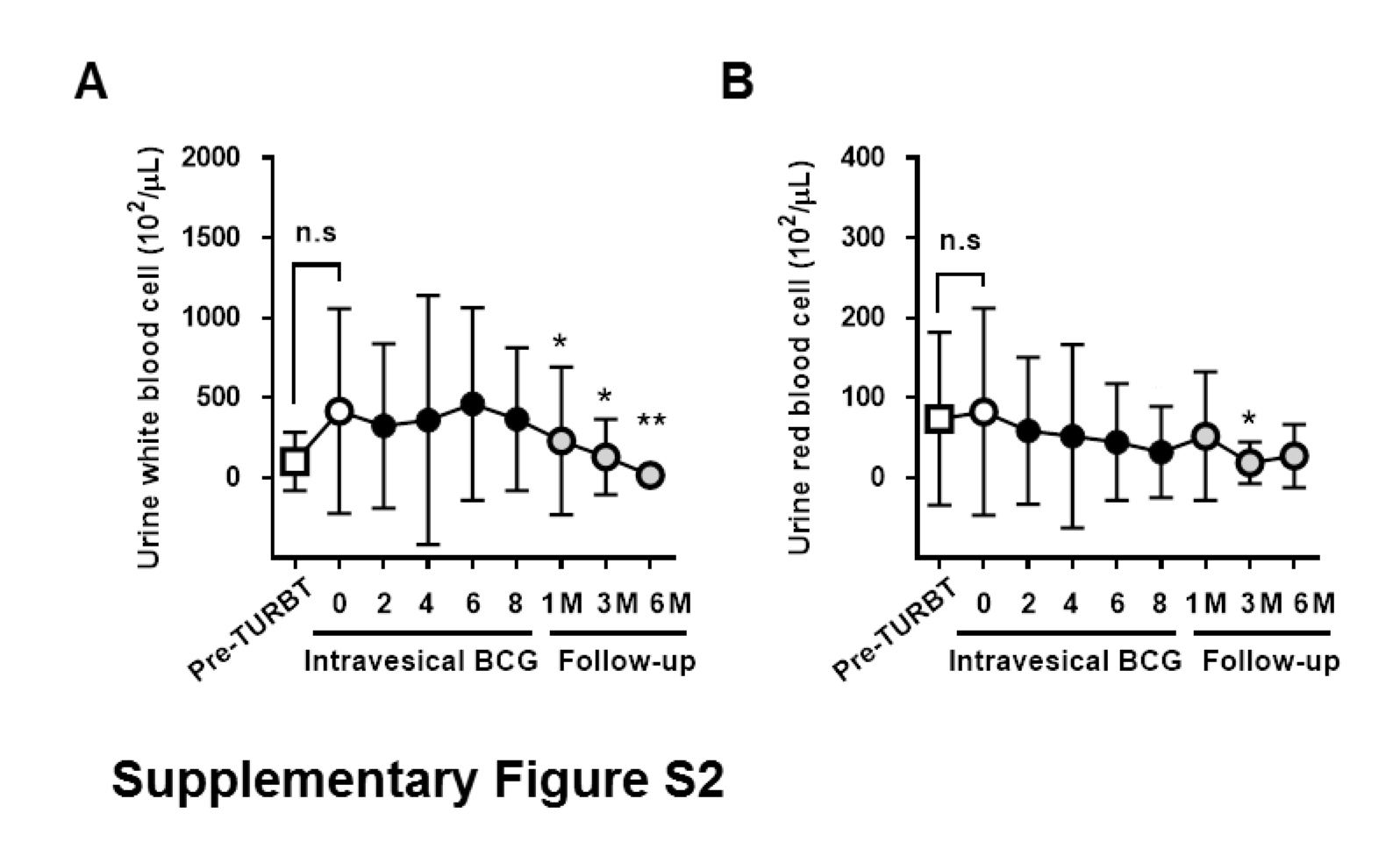

Supplement: Supplementary file 1 [file diseases-07-00044-s001.zip › Supplementary Figure S2.tif]

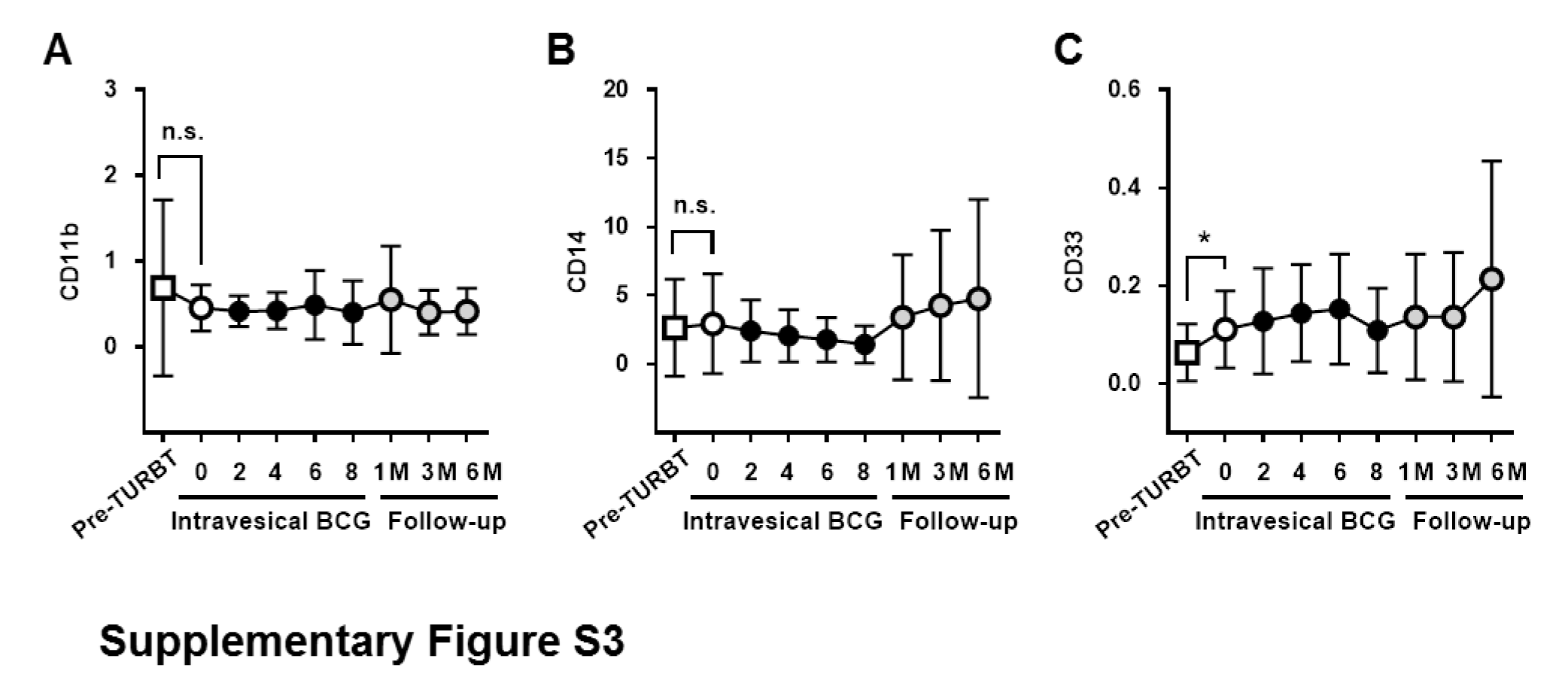

Supplement: Supplementary file 1 [file diseases-07-00044-s001.zip › Supplementary Figure S3.tif]

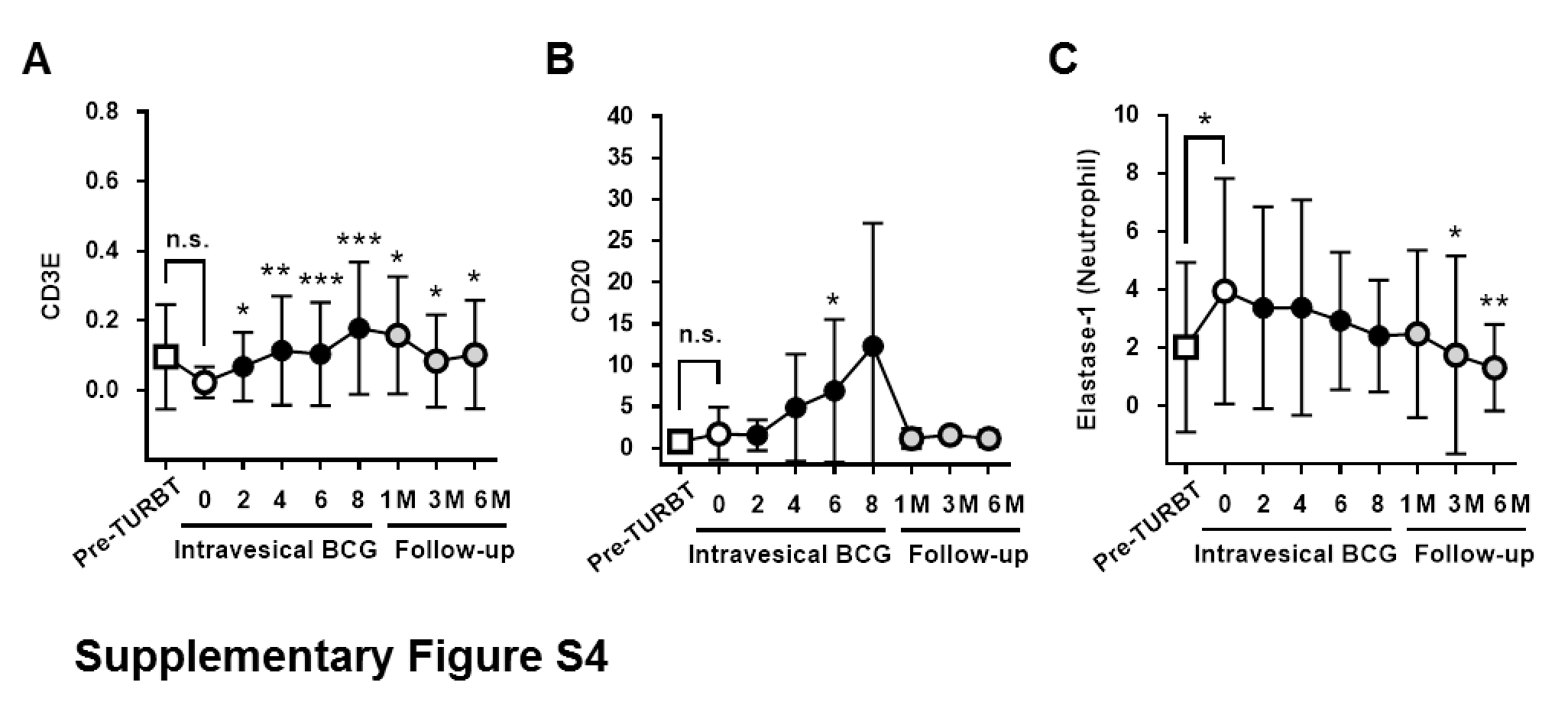

Supplement: Supplementary file 1 [file diseases-07-00044-s001.zip › Supplementary Figure S4.tif]
